# Improved feasibility of handheld optical coherence tomography in children with craniosynostosis

**DOI:** 10.1038/s41433-024-03118-w

**Published:** 2024-05-24

**Authors:** Sohaib R. Rufai, Vasiliki Panteli, Robert H. Henderson, Catey Bunce, Irene Gottlob, Mervyn G. Thomas, Frank A. Proudlock, Richard Bowman, Noor ul Owase Jeelani

**Affiliations:** 1 Clinical and Academic Department of Ophthalmology, Great Ormond Street Hospital for Children NHS Foundation Trust and UCL Great Ormond Street Institute of Child Health, London, UK; 2 The University of Leicester Ulverscroft Eye Unit, Leicester Royal Infirmary, Leicester, UK; 3 Craniofacial Unit, Great Ormond Street Hospital for Children NHS Foundation Trust and UCL Great Ormond Street Institute of Child Health, London, UK; 4 Clinical Trials Unit, The Royal Marsden NHS Foundation Trust, London, UK

**Keywords:** Biomarkers, Medical research, Optic nerve diseases, Tomography

Craniosynostosis is characterised by the premature fusion of the cranial sutures and is often associated with intracranial hypertension (IH) [1]. This can damage the brain and vision if unaddressed [2]. Direct monitoring of intracranial pressure (ICP) is invasive and carries risks. Optical coherence tomography (OCT) is non-invasive and has demonstrated good potential as a surrogate measure of ICP in craniosynostosis [3, 4]. Our group found good feasibility of handheld OCT in 50 children with craniosynostosis, demonstrating 86% success rate in at least one eye and 76% in both eyes [5]. The main reason for non-acquisition was loss of interest [5]. Here, we report improved feasibility in a larger craniosynostosis cohort.

This was a cross-sectional study of 85 children with craniosynostosis recruited from Great Ormond Street Hospital for Children, London, UK, between 12th November 2020 and 21st July 2022. Inclusion criteria were as follow: (i) diagnosis of craniosynostosis; (ii) age 0–18 years; (iii) availability of parent/guardian to provide consent. An electronic medical record (Epic Systems) was used to highlight eligible patients in advance, track locations in real time, send secure messages to colleagues and avoid long waits.

All handheld OCT examinations were performed by the lead investigator (SRR) using the Envisu C2300 device (Leica Microsystems) (Fig. 1). Recruitment success rate was recorded. Handheld OCT imaging success rates were recorded at the following levels: (i) at least one optic nerve head image acquired, of analysable quality; (ii) bilateral optic nerve head images acquired, of analysable quality. Analysable quality was defined as an optic nerve head tomogram wherein the edges of the disc margins and the cup profile, including its lowermost point, were clearly visualised. Full details of our recruitment and handheld OCT methodology are detailed in our previous feasibility paper [5].

**Fig. 1:**
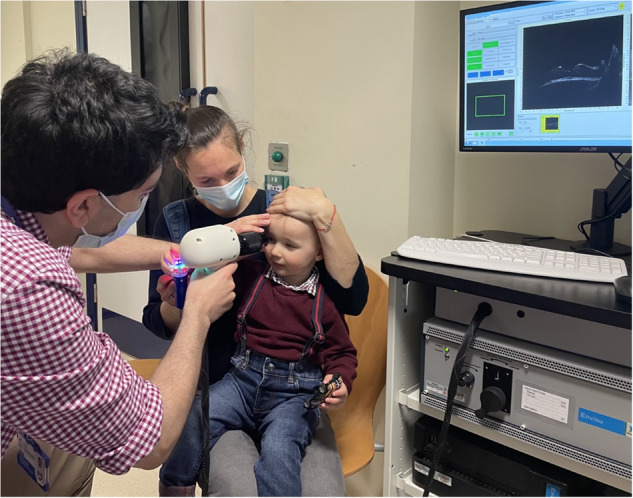
Handheld OCT examination in young child with craniosynostosis. Photograph taken with parental consent. *Source:* Rufai SR*,* et al. [5].

Out of 85 children approached for recruitment, 85 (100%) were successfully recruited. Overall median age was 66 months (range: 18–183; interquartile range [IQR]: 43–90), 49 (58%) were male and 31 (36%) had syndromic craniosynostosis. Six children (8%) were imaged whilst asleep (anaesthetised, *n* = 3; napping, *n* = 3) and 79 (92%) were awake. At least one image of analysable quality was successfully obtained in 81 children (95%), and bilateral images in 72 children (85%). The successful group were older (median age: 67 months; range: 19–183; IQR: 43–90) than the unsuccessful group (median age: 39 months; range: 18–116; IQR: 24–68). Of the unsuccessful group, all four (100%) were males who lost interest, and two patients (50%) had syndromic craniosynostosis.

This study demonstrated improved feasibility compared to our previous study [5] Whilst this could be due to chance, we believe it is more likely due to avoiding long patient waits by optimising the use of Epic, and improved experience of the lead investigator (SRR), possibly suggesting a ‘learning curve’ in handheld OCT examination of this patient population. This cohort was slightly older than the previous cohort (median age: 51 months, range: 2–157; IQR: 37–74), which may have contributed to improved success rates. In conclusion, handheld OCT is acceptable and feasible in children with craniosynostosis.

## Data Availability

The data that support the findings of this study are available from the corresponding author upon reasonable request.
